# Performance Validation of a New Portable Ophthalmic Ultrasound Device

**DOI:** 10.7759/cureus.80711

**Published:** 2025-03-17

**Authors:** Dhanush P Pandya, Kiruthika Kannan, Kannan Venkataraju, Saarang Hansraj, Rajeev Reddy Pappuru, Taraprasad Das, Ravichandran Kondusamy, Jayasankar Sagadevan, Akash Belenje

**Affiliations:** 1 Srimati Kanuri Santhamma Centre for Vitreo-Retinal Diseases, Anant Bajaj Retina Institute, Kallam Anji Reddy Campus, LV Prasad Eye Institute, Hyderabad, IND; 2 Research and Development, Appasamy Associates Private Limited, Chennai, IND

**Keywords:** b-scan, imaging, portable ultrasonography, retina, ultrasonography (usg)

## Abstract

Purpose

This study aimed to compare a new indigenous portable ophthalmic ultrasound device with an industry-standard multinational device for its accuracy, precision, and potential use in home care.

Methods

Axial and transverse images were compared for the same eye at the same visit using the India-made and multinational-made industry-standard devices for several common and rare vitreoretinal conditions in a large referral center in India. All images were obtained by the same optometrist on the same day using the India- and multinational-made devices in that sequence. Two third-year retina fellow investigators masked to the diagnosis and device analyzed the images for inter- and intra-observer accuracy of clinical diagnosis. The clinical diagnosis was compared with a senior vitreoretinal faculty member.

Results

The study included 118 eyes scanned once with each device. The overall accuracy was 88.9% for the portable India-made device and 85.5% for the multinational-made device. The inter-observer agreement and kappa were the same for the India-made and multinational-made devices; these were 91.52% and 0.83, respectively. The intra-observer agreement for various diagnoses for grader 1 was 95% (Cohen's kappa: 0.89) and for grader 2 was 93.3% (Cohen's kappa: 0.86).

Conclusion

The India-made portable ophthalmic ultrasound device was non-inferior to the multinational-made device in clinical utility. Its advantages were portability, lesser cost, and ease of use, which make the device more suitable for home eye care.

## Introduction

Ultrasonography is an important investigation for diagnosing several ocular and orbital diseases. Introduced in the 1950s and further developed in the next decade, ophthalmic ultrasonography is today's standard of eye care diagnoses[1]. It uses high-frequency sound waves transmitted from a probe (piezoelectric transducer) into the eye. After striking intraocular structures, the sound waves are reflected to the probe and converted into an electrical signal for image reconstruction and evaluation of the eye. There are two basic modes: the A-scan (amplitude scan) and the B-scan (brightness scan). In A-scan, ultrasonography is a one-dimensional scan of the eye that gives details of its length[1-4]. B-scan ultrasonography is a two-dimensional cross-section of the eye and orbit used for posterior segment evaluation of the eye with media opacities [1,2,5,6]. It is non-invasive, is easily available, and produces high-resolution images, and the results are reproducible[7].

According to a global marketing survey [8], the DGH (Exton, Pennsylvania, United States), Accutome (Keeler, Berkshire, United Kingdom), Nidek (Gamagori, Japan), and Micro Medical Devices (Calabasas, California, United States) are the current international market leaders for ultrasonography machines. In India, Appasamy Associates (Chennai) is a leading manufacturer of ophthalmic ultrasonograms. While table-top ophthalmic ultrasound systems are commonly used, reaching people closer to their residences is a growing necessity, particularly felt during the SARS-CoV-2 pandemic[9]. This is possible with a portable B-scan system that is also lightweight and easy to use. Appasamy Associates has developed one such portable system in India. The hardware of the machine is imported, and the assembly takes place in India. The software, along with its intellectual property (IP), is also developed and based in India. 

In this study, we evaluated the potential clinical utility of an indigenous portable ophthalmic ultrasound device in comparison to an industry-standard system. Our analysis focused primarily on B-scan imaging and the device's ability to detect various posterior segment pathologies. By comparing these features, we aimed to determine whether the portable device could serve as a viable alternative in clinical practice.

## Materials and methods

The study was approved by the Institutional Review Board (IRB) of LV Prasad Eye Institute (approval number: LEC-BHR-P-07-23-1071) on July 7, 2023, and was conducted adhering to the tenets of the Declaration of Helsinki. Written and Informed consent was obtained from all patients undergoing the scans.

Study design

This prospective observational study was conducted from July 2023 to June 2024 at LV Prasad Eye Institute in Hyderabad, India. The study was designed to compare the images of the eyes of patients obtained from two devices by the same sonographer in similar examination conditions. The industry-standard machine was Accutome by Keeler (B-Scan Plus (model number: 24-6100)), while the India-made portable B-scan machine was from Appasamy Associates (prototype).

Study population

This study included 118 patients who were referred from the retina service for B-scan ultrasonography due to media opacity obstructing accurate posterior segment evaluation.

Sample size estimation

Suppose we assume the accuracy of a standard (multinational-made) device to be 100% and the expected difference of 10% in accuracy with the new portable (India-made) device with an alpha of 0.05% (confidence limit of 95%) and a power of 90%, the required sample size would be 95. We recruited 118 people for this study.

Examination technique

Techniques were similar in both devices and were performed by the same certified sonographer. Briefly, it was as follows: during the examination, the patient was seated in the primary position between two instruments, and a B-scan was conducted while the patient remained seated. Transverse and axial scans were performed for all patients. A horizontal-directed marker pointing towards the nose was utilized to scan the superior and inferior quadrants, and a vertical-directed marker pointing towards the 12 o'clock position was used to scan nasal and temporal quadrants. In axial scans, the marker's direction was oriented horizontally. Before placing the probe, ultrasound conduction gel (Aqua Gel, Hypoallergenic Ultrasound Transmission Gel, IR Surgicals, Hyderabad, India) was applied to the eyelids, and scans were performed with closed eyelids. Patients were instructed to look in the direction corresponding to the examination area; for example, if the temporal area of the right eye needed examination, patients were asked to look in a dextro-version gaze. The probe was then placed perpendicularly for transverse and longitudinal scans. Axial scans were obtained with the patient in the primary position. 

To standardize image acquisition, power was kept fixed at 100%, and initial gain was set at 80 dB. Gain was later fine-tuned as per the findings.

Special examination techniques were used to assess some retinal conditions which included studying the aftermovement, convection motion, topographic dimensions, and reflectivity.

Masking and image analysis

Two-masked final-year retina fellows (a three-year program) analyzed the images (all the clinically relevant information were removed). The study measured inter-observer and intra-observer agreements. Intra-observer agreement was evaluated by reanalyzing a subset of 60 images selected by a senior vitreoretinal faculty member, 15 days apart to validate consistency in assessments. The clinical diagnoses were compared with a senior vitreoretinal faculty member for validation.

Machine details

**Figure 1:**
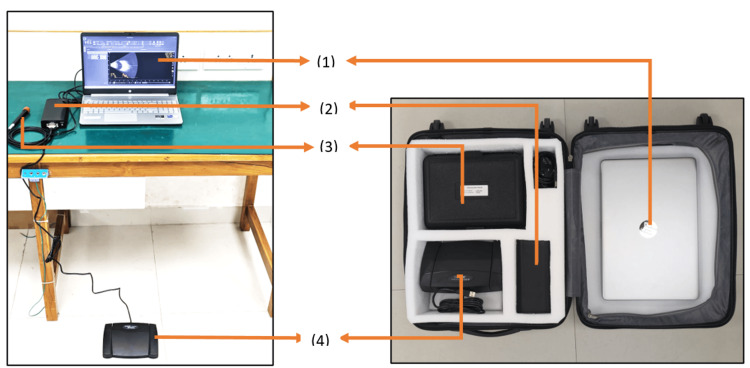
India-made B-scan: (left) assembled for use and (right) assembled for transportation (1) Laptop. (2) Console. (3) B-scan probe. (4) Footswitch with image, video capture, and gain adjustment

The portable India-made B-scan device consists of a console, a 10 MHz ultrasound probe, a foot pedal, and a specifically configured 13.5-inch laptop housed in a suitcase that can be wheeled easily. The console is electrically charged (250 watts), and the probe, the foot pedal, and the laptop are attached to the console (Figure 1).

The multinational-made table-top B-scan consisted of a B-scan ultrasound probe, probe holder, footswitch, wireless mouse, CD container, software, and user manual.

Table 1 lists the differentiating features between the India-made (Appasamy) and multinational-made (Accutome) devices.

**Table 1:** Differentiating features between Appasamy (India) and Accutome (multinational) devices IEC: International Electrotechnical Commission; USB: Universal Serial Bus; USG: ultrasonography

Feature	Appasamy device	Accutome device
Components included	Console, 10 MHz ultrasound probe, foot pedal, 13.5-inch laptop in a suitcase	12 MHz ultrasound probe, footswitch, probe holder, cordless mouse
Laptop	Included in the device	Not included with the device
Ultrasound probe	10 MHz	12 MHz
Probe specifications	Length: 4.6 inches. Diameter: 0.62 inch. Cable length: 6 feet. Weight: 0.05 kg (50 g). D-type connection	Length: 7 inches. Diameter: 1.25 inch. Cable length: 6 feet. Weight: 0.17 kg. USG type A connection
Portability	Wheeled suitcase for easy mobility	Lightweight. Table-top
Safety	IEC 60601. IEC certified. Type b medical device	IEC 60601. IEC certified. Type b medical device
Cost	Less expensive (two-thirds of the multinational-made device tested in the study)	More expensive than the India-made device
Footswitch	8×5.5×1.5 inches 0.915 kg USB type A connection	4×3.25×1.25 inches 0.153 kg USG type A connection
Probe holder	10×7.5×3 inches 0.4 kg	7×4.3×2.09 inches 0.34 kg
Computer system	Provided	Not provided

Statistical analysis 

The SAS software (Version 3.81, SAS Inc., Cary, North Carolina, United States) was used for the analysis. Intra-observer and inter-observer analysis used Cohen's kappa analysis. A two-tailed t-test was done to compare quantitative data. A p-value of <0.05 was considered statistically significant (alpha=0.05).

## Results

This study included 118 eyes of 118 patients. The mean age was 47.63 years and included 80 male and 38 female participants. The common conditions were vitreous hemorrhage (n=27), retinal detachment (n=18), and choroidal detachment (n=15) (Table 2).

**Table 2:** Diagnosis of participants

Pathology	n
Vitritis/vitreous hemorrhage/endophthalmitis	27
Retinal detachment	18
Choroidal detachment	15
Silicon oil-filled oil	8
Normal	6
Melanoma	6
Emulsified oil	6
Asteroid hyalosis	5
Posterior vitreous detachment	5
Air-filled/gas-filled globe	5
Retinochoroidal coloboma	3
Posterior staphyloma	3
Retinoblastoma	2
Optic nerve head drusen	2
Sub-Tenon's fluid	2
Optic disc cupping	1
Status post scleral buckle	1
Hemangioma	1
Intraocular foreign body	1
Phthisis bulbi	1
Total	118

The overall accuracy was 88.9% for the portable India-made device and 84.7-86.4% (average 85.5%) for the multinational-made device tested in this study. There was no statistical difference between the readers and the machines (p=0.31; two-tailed paired samples t-test) (Table 3).

**Table 3:** Overall accuracy in the diagnosis Machine 1: multinational-made device; machine 2: India-made device

Machines	Data type	Reader 1	Reader 2	P-value
Machine 1	Correctly diagnosed	102/118 (86.4%)	100/118 (84.7%)	0.710
Machine 2	Correctly diagnosed	105/118 (89%)	105 (89%)	0.999
Machine 1	Incorrectly diagnosed	16/118 (13.6%)	18/118 (15.3%)	0.270
Machine 2	Incorrectly diagnosed	13/118 (11%)	13/118 (11%)	0.999

The agreement and Cohen's kappa were 91.5% and 0.83, respectively, in either device between the two masked readers. Between the three common disorders and multinational-made and India-made devices, the agreement was high (100% and 93.3%) for choroidal detachment and relatively low for vitreous hemorrhage/endophthalmitis (81.5% in both devices). Cohen's kappa was 1.0, 0.86, and 0.63, respectively (Table 4).

**Table 4:** Agreement based on various diagnoses and individual kappa

Diagnosis	Multinational-made device		India-made device	
	Agreement (%)	Cohen's kappa	Agreement (%)	Cohen's kappa
Vitritis/vitreous hemorrhage/endophthalmitis	81.48%	0.63	81.48%	0.63
Retinal detachment	94.4%	0.88	88.8%	0.77
Choroidal detachment	100%	1	93.3%	0.86
Silicon oil-filled oil	100%	1	100%	1
Normal	100%	1	83.3%	0.67
Melanoma	83.3%	0.67	83.3%	0.67
Emulsified oil	100%	1	100%	1
Asteroid hyalosis	100%	1	100%	1
Posterior vitreous detachment	80%	0.61	100%	1
Air-filled/gas-filled globe	80%	0.61	100%	1
Retinochoroidal coloboma	100%	1	100%	1
Posterior staphyloma	100%	1	100%	1
Retinoblastoma	100%	1	100%	1
Optic nerve head drusen	50%	-	100%	1
Sub-Tenon's fluid	100%	1	100%	1
Optic disc cupping	100%	1	100%	1
Status post scleral buckle	100%	1	100%	1
Hemangioma	100%	1	100%	1
Intraocular foreign bodies	100%	1	100%	1
Phthisis bulbi	100%	1	100%	1
Total agreement	91.52%	0.83	91.52%	0.83

The intra-observer kappa was 0.89 and 0.86 for the masked graders 1 and 2, respectively (95% agreement, 93.3% agreement) (Table 5).

**Table 5:** Intra-observer agreement for the readings taken 15 days apart of the same scans

Reader 1: after 15 days		Reader 2: after 15 days	
Agreement after 15 days	Kappa	Agreement after 15 days	Kappa
57/60 (95%)	0.89	56/60 (93.3%)	0.86

**Figure 2:**
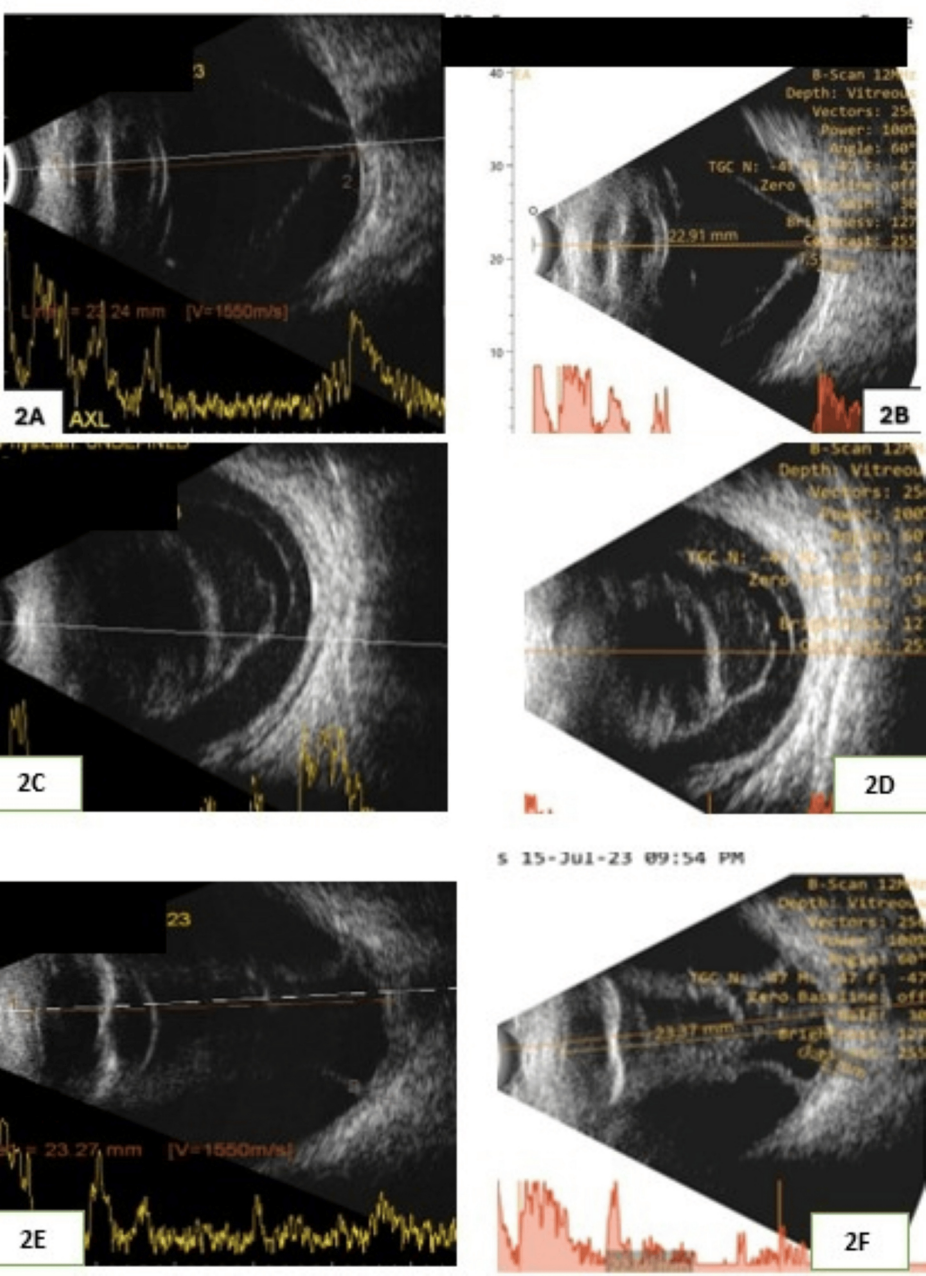
B-scan ultrasound in two ultrasonograms: (left) multinational-made device image and (right) India-made device image Top: retinal detachment. Middle: vitreous hemorrhage. Bottom: choroidal detachment

**Figure 3:**
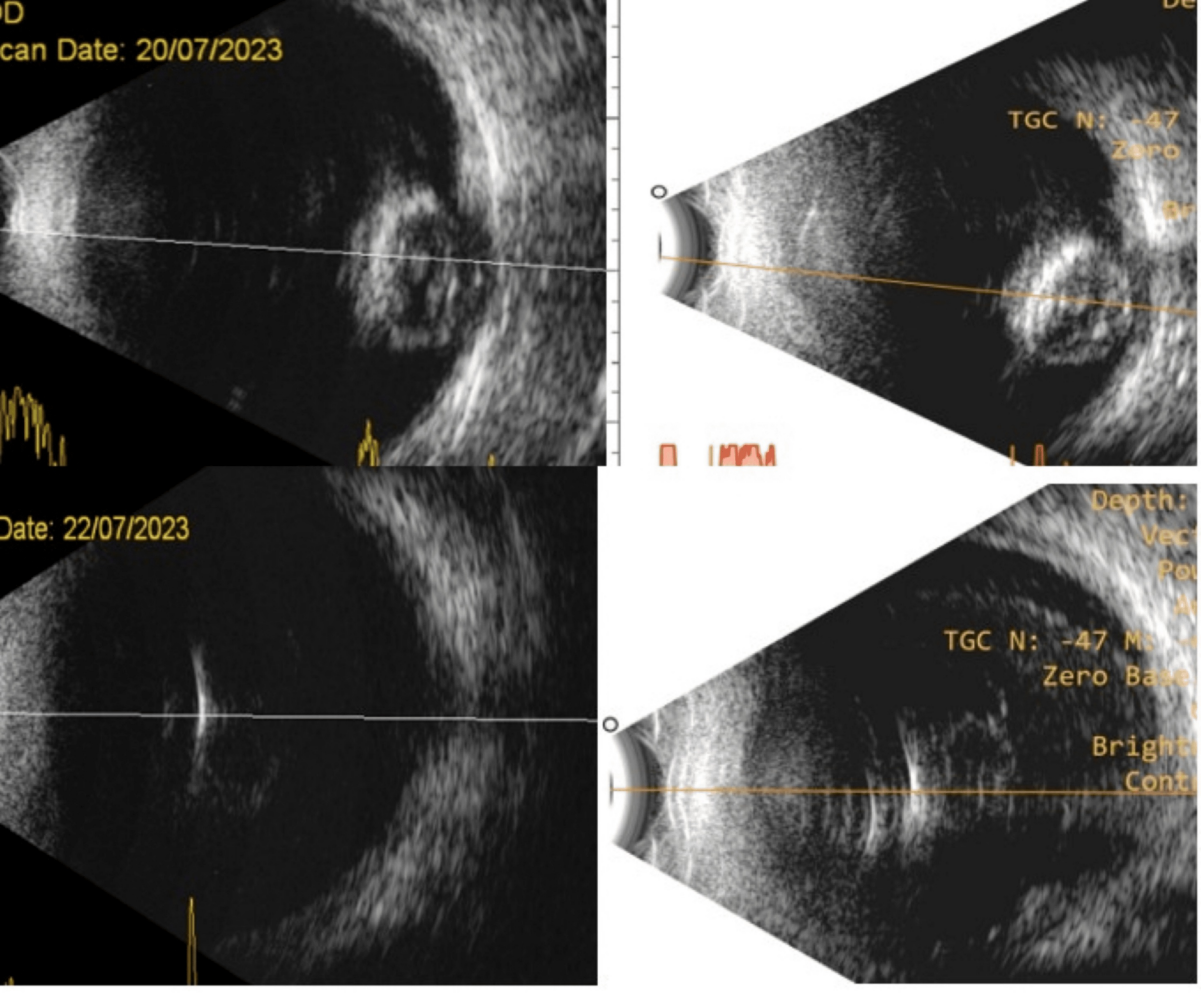
B-scan ultrasound in two ultrasonograms: (left) multinational-made device image and (right) India-made device image Top: Nucleus drop. Bottom: Intraocular foreign body

We measured the difference in axial length between the two machines. In this cohort, 98 of 118 (83%) eyes had a difference of less than 0.75 mm between the two devices. But it was not significant. The t-test revealed a p-value of 0.062 (more than the pre-decided value of p=0.05). Hence, the difference in axial length was insignificant (Figure 2 and Figure 3). 

## Discussion

Ophthalmic ultrasonography is an essential component of comprehensive eye care. A portable and good-quality ultrasonographic device has certain unique advantages over table-top devices. It could be used as a point of care for people who can't travel and for less abled people, including people living in old-age homes. Also, point-of-care ultrasonography (POCUS) is not a novel concept; its diagnostic advantages are well-established across various medical fields[9,10]. Few studies have highlighted its potential in the rapid assessment of ocular trauma, retinal detachment, and other sight-threatening conditions, reinforcing its role as a valuable bedside tool[11,12]. Given its non-invasive nature, portability, and ability to provide real-time imaging, further research and integration into routine emergency ophthalmic care could significantly enhance patient outcomes.

This study showed that an India-made portable ultrasonographic device is not inferior to a multinational-made device in diagnosing posterior segment diseases. The combination of A- and B-scan and reduced cost are added advantages. Thus, it is particularly useful in economically challenged countries and regions of the world.

Earlier studies have compared the diagnostic confidence and image quality of a hand-held ophthalmic ultrasound device with standard ophthalmic ultrasound machines (Association for Research in Vision and Ophthalmology (ARVO) presentations 2021 and 2023 [13,14]), but not as much the diagnostic capabilities. With good inter-observer (91.5%) and intra-observer (93.3-95%) agreement and good inter-observer kappa (0.83), the India-made device is a reliable system for use in ophthalmic practice.

As noted in the study done by Nathe et al. [15], agreement in retinal detachment diagnosis between the portable device and conventional device was 97.5%, and Cohen's kappa was 0.950. As compared to our study, the agreement between two readers for the diagnosis of retinal detachment was 88.8% in the portable machine with a kappa of 0.77. Our study employed a different methodology, where we not only compared the agreement between readers but also assessed their agreement with the clinical diagnosis. Given this approach, the kappa and agreement values are slightly lower, which is an expected outcome.

In our study, accuracy in diagnosing retinal detachment was 88.8%, which is notably higher than the 78% reported in studies involving open globe injuries[16]. However, our study included all types of rhegmatogenous retinal detachment and not just post-open globe injury.

In this study, we were able to determine that the accuracy of the diagnosis of vitreous opacity without relevant clinical information was about 81.5%. This highlights the inherent limitations of B-scan ultrasonography as a standalone diagnostic tool, particularly in differentiating between vitreous hemorrhage and inflammatory vitreous opacities. The utility of POCUS for endophthalmitis has already been described in the literature[17]. The ability to perform real-time, bedside imaging makes POCUS valuable in resource-limited settings.

A portable and hand-held ophthalmic ultrasonogram is a novel concept. It provides a much-needed point of care for people who can't or need not travel to eye care facilities. The portable B-scan device we tested is not new in the world market but the first of its kind in India. While it is not inferior to another standard-of-care multinational-made device, it is undoubtedly less expensive and more affordable. It meets all technical safety parameters and is easy to assemble and operate.

Study limitations

The study could not be masked to the optometrist performing the B-scan which can lead to potential bias during image acquisition. Our study was confined to vitreoretinal pathology only; it didn't include primary orbital pathology and didn't study the aftermovement of the membrane which can provide further insights. Other limitations include the relatively small sample size and the focus on only a subset of ophthalmic pathologies which may limit the generalizability of the findings to other ocular conditions.

## Conclusions

The India-made portable ultrasonographic device is as accurate as the industry-standard device in diagnosing common vitreoretinal conditions and measuring the axial length of the eye which is essential for effective eye health management. Its compact size and affordable cost allow it to be conveniently transported, making it an excellent option for point-of-care diagnosis.

This device is particularly beneficial in low-resource countries and remote settings where access to imaging equipment may be limited. Its portability and ease of use enable healthcare providers to offer essential diagnostic services in underserved areas, significantly enhancing access to eye care for populations in need without compromising on quality.
